# Brain-inspired Predictive Coding Improves the Performance of Machine Challenging Tasks

**DOI:** 10.3389/fncom.2022.1062678

**Published:** 2022-11-16

**Authors:** Jangho Lee, Jeonghee Jo, Byounghwa Lee, Jung-Hoon Lee, Sungroh Yoon

**Affiliations:** ^1^Department of Electrical and Computer Engineering, Seoul National University, Seoul, South Korea; ^2^Institute of New Media and Communications, Seoul National University, Seoul, South Korea; ^3^CybreBrain Research Section, Electronics and Telecommunications Research Institute (ETRI), Daejeon, South Korea; ^4^Interdisciplinary Program in Artificial Intelligence, Seoul National University, Seoul, South Korea

**Keywords:** brain-inspired learning, biologically plausible learning, deep learning, backpropagation, predictive coding

## Abstract

Backpropagation has been regarded as the most favorable algorithm for training artificial neural networks. However, it has been criticized for its biological implausibility because its learning mechanism contradicts the human brain. Although backpropagation has achieved super-human performance in various machine learning applications, it often shows limited performance in specific tasks. We collectively referred to such tasks as *machine-challenging tasks* (MCTs) and aimed to investigate methods to enhance machine learning for MCTs. Specifically, we start with a natural question: *Can a learning mechanism that mimics the human brain lead to the improvement of MCT performances?* We hypothesized that a learning mechanism replicating the human brain is effective for tasks where machine intelligence is difficult. Multiple experiments corresponding to specific types of MCTs where machine intelligence has room to improve performance were performed using predictive coding, a more biologically plausible learning algorithm than backpropagation. This study regarded incremental learning, long-tailed, and few-shot recognition as representative MCTs. With extensive experiments, we examined the effectiveness of predictive coding that robustly outperformed backpropagation-trained networks for the MCTs. We demonstrated that predictive coding-based incremental learning alleviates the effect of catastrophic forgetting. Next, predictive coding-based learning mitigates the classification bias in long-tailed recognition. Finally, we verified that the network trained with predictive coding could correctly predict corresponding targets with few samples. We analyzed the experimental result by drawing analogies between the properties of predictive coding networks and those of the human brain and discussing the potential of predictive coding networks in general machine learning.

## 1. Introduction

The human brain has an intricate and heterogeneous structure that consists of a high recurrent and nonlinear neural network (Felleman and Van Essen, 1991; Friston, 2008; Bertolero et al., 2015). It is commonly understood that the learning system of the human brain operates on the synaptic plasticity mechanism (Hebb, 2005), wherein the modulation in synaptic weights varies according to the intrinsic or extrinsic stimuli (Power and Schlaggar, 2017). Specifically, neural plasticity regulates the process of synaptic transmission as a fundamental property of neurons (Citri and Malenka, 2008; Mateos-Aparicio and Rodŕıguez-Moreno, 2019). Based on this property, the neuronal responses to sensory stimuli enable the robust recognition (Ohayon et al., 2012; Denève et al., 2017; Geirhos et al., 2017; Wardle et al., 2020) and noise-resistance learning (Suzuki et al., 2015; Perez-Nieves et al., 2021) in human perception.

Based on the human brain architecture, artificial neural networks (ANNs) were suggested to simulate the pattern of the human decision-making process for recognition tasks. Rumelhart et al. (1986) introduced the backpropagation algorithm that adjusts the network parameters to achieve reliable performance. Backpropagation iteratively updates the network parameters relying on the error signal generated at the end of the network between the produced output and the desired output. In the last decade, with the benefits of backpropagation (Rumelhart et al., 1986), ANNs have exceeded human-level performance on classification, segmentation, and detection (He et al., 2016; Dosovitskiy et al., 2020). However, learning ANNs with backpropagation have been criticized for their biological implausibility, wherein its behavior conflicts with the activity of real neurons in the human brain (Akrout et al., 2019; Illing et al., 2021). First, the human brain operates according to *neural plasticity*, which indicates the capability for modifying neural circuit connectivity or degree of interaction (Neves et al., 2008). Second, global error-guided learning requires the forward weight matrices to propagate the error signal flow to the lower layer, that is *weight transport problem* (Grossberg, 1987). Multiple learning algorithms have been proposed to alleviate the previously mentioned issues based on strong constraints of backpropagation and reinforce its biological plausibility (Liao et al., 2016; Lillicrap et al., 2016; Whittington and Bogacz, 2017; Woo et al., 2021; Dellaferrera and Kreiman, 2022). This study explored the predictive coding network (Whittington and Bogacz, 2017) among the various biologically plausible learning and its characteristics.

A predictive coding network (Whittington and Bogacz, 2017) was introduced to resolve the biological limitations of backpropagation depending on the hierarchically organized visual cortex of the human brain (Rao and Ballard, 1999; Friston, 2008). With respect to biological plausibility, a predictive coding network concentrates on local and Hebbian plasticity by minimizing the prediction errors between expected and actual inputs (Rao and Ballard, 1999; Millidge et al., 2020). The learning mechanism of the predictive coding network is different from that of backpropagation, which updates the network parameters using only one error derived from the last layer (Rumelhart et al., 1986). Predictive coding is regarded as a local learning algorithm because its learning is performed with local error nodes and global error nodes. A learning network with predictive coding approximates the learning dynamics of backpropagation (Whittington and Bogacz, 2017) and can also be expanded to arbitrary computational graphs (Millidge et al., 2020). Multiple works (Han et al., 2018; Wen et al., 2018; Choksi et al., 2021) inspired by the property of prediction itself have been proposed, and some studies (Choksi et al., 2021; Salvatori et al., 2021) demonstrated that the potential of the predictive manner related to human perception.

However, despite the remarkable accomplishment of ANN architectures and their learning algorithms, there remains a performance gap between machine and human intelligence in some applications. We collectively refer to these tasks as *machine-challenging tasks* (MCTs); MCTs are difficult for machine intelligence while easy for human intelligence. This study considers the representative MCTs as incremental learning, long-tailed recognition, and few-shot learning (Hassabis et al., 2017). A more detailed definition and explanation of MCTs will be presented in Section 2.2. Humans progressively and ceaselessly acquire new knowledge and preserve it by virtue of the hippocampus (Preston and Eichenbaum, 2013). The primary function of the hippocampus is that it enables long-term memory of the spatial and sequential order from the human experience (Bird and Burgess, 2008; Davachi and DuBrow, 2015). This property makes the human intelligence exhibits robust and performs better than machine intelligence (Goodfellow et al., 2014; Zhou and Firestone, 2019; Liu et al., 2021). Meanwhile, ANNs trained with backpropagation tend to forget what it learned when it learns new information, that is *catastrophic forgetting* (McCloskey and Cohen, 1989; French, 1999; Goodfellow et al., 2013). As another example, machine intelligence shows unsatisfactory performance under limited or imperfect training data recognition (De Man et al., 2019; Liu et al., 2019a). When training ANNs for classification tasks in a long-tail scenario, the classifier can be easily biased toward the majority classes that contain the most data and show poor performance in minority classes (Johnson and Khoshgoftaar, 2019). These phenomena result from the fundamental differences in visual processing between the brain and ANNs (Xu and Vaziri-Pashkam, 2021). Inspired by Hassabis et al. (2017), we hypothesized that the closer the learning algorithm is to the human brain, the more effective it is for the MCTs.

Similar to our assumption on the MCTs, the learning algorithms inspired by the brain are consistently studied to reduce the performance gap between machine intelligence and human intelligence based on human's various attributes. In terms of human learning mechanisms, a spiking neural network (SNN) is considered a promising solution to replicate the neural processing process of the brain. On the other hand, recent studies reported that the neural network trained biologically plausible manner embodies specific memory functions in the human memory system. Therefore, based on previous studies, we speculated that predictive coding has other latent properties. This study aimed to discover hidden properties and extend the scope of predictive coding to MCTs. Contrary to the conventional solutions for the MCTs, our study focused on the predictive coding algorithm itself employed for the optimization of the network parameters. In incremental learning, it is confirmed that predictive coding better reveals the plasticity-stability property and enables faster adaptation to new tasks than backpropagation. In long-tailed recognition, it reduces the classification bias problem of minority classes.

This paper is organized as follows: In Section 2, the predictive coding network is briefly reviewed. In Section 3, the experiments on incremental learning based on a predictive coding network are presented. In Section 4, the experiments on limited data recognition based on a predictive coding network, such as long-tailed recognition and few-shot learning, are described. In Section 5, we discuss why predictive coding network improves the performance of MCTs. In Section 6, related work to help understand our paper is presented. In Section 7, we conclude the paper with limitations and a summary.

Our contributions can be summarized as follows:

- The study characterized the MCTs, which are easy for human intelligence and difficult for machine intelligence, in machine learning fields and proposed a hypothesis that the brain-inspired learning algorithm improves the performance of MCTs.
- Predictive coding, a biologically plausible learning algorithm, was adopted for MCTs, such as incremental learning and limited data recognition. In addition, extensive experiments were performed by reimplementing the learning with backpropagation with predictive coding.
- The effect of learning algorithms close to brain learning on MCTs in terms of neuroscience was presented. Mainly, the experimental results were analyzed with respect to the plasticity-stability dilemma and interplay between the hippocampus and prefrontal cortex.

## 2. Related Work

### 2.1. Biologically Plausible Learning

The backpropagation algorithm (Rumelhart et al., 1986), which simulates the properties of the human brain, has achieved excellent progress in various machine learning tasks. The algorithm calculates the global error by comparing the predicted outputs and the actual targets at the network's end to achieve an objective. Then, it propagates the error signal to the front of the network to update parameters. Although backpropagation is the most popular learning algorithm for ANNs, it is often regarded as a biologically implausible algorithm from a neuroscience perspective. The main reason is that backpropagation does not operate following the local synaptic plasticity (Takesian and Hensch, 2013; Mateos-Aparicio and Rodŕıguez-Moreno, 2019) as a fundamental property of the nervous system. Synaptic plasticity refers to the ability to reorganize structures or connections by intrinsic or extrinsic stimuli. Another reason is that the backpropagation requires a copy of the weight matrices to transfer backward error signal (Grossberg, 1987). However, retaining synaptic weights on each neuron is impractical in the human brain. So, Lillicrap et al. (2016) replaced the backward weight matrices with fixed random weights to avoid those problems. Liao et al. (2016) reported that the signs of backward weight matrices were important, and when the signs between the forward and backward matrices were concordant, the same or better performance could be achieved. Furthermore, various learning algorithms have been proposed to reinforce biological plausibility while maintaining the classification performance (Lee et al., 2015; Whittington and Bogacz, 2017; Ahmad et al., 2020; Lindsey and Litwin-Kumar, 2020; Pogodin and Latham, 2020). Among them, predictive coding, based on the predictive process of the brain, was suggested to achieve better biologically plausible properties than the backpropagation algorithm and achieved comparable performance to the backpropagation on arbitrary computational graphs (Whittington and Bogacz, 2017).

### 2.2. Machine Challenging Tasks (MCTs)

ANNs have achieved comparable or superior performances to humans by backpropagation in visual recognition (Russakovsky et al., 2015; Geirhos et al., 2017). However, ANNs have unsatisfactory performance in certain tasks regarded as simple and easy for human intelligence (Goodfellow et al., 2013; Snell et al., 2017; Cao et al., 2019). As detailed in Section 1, these types of tasks as MCTs (e.g., incremental learning, long-tailed recognition, and few-shot recognition).

Humans ceaselessly take new information from multiple sensory organs and reorganize it in the brain (Felleman and Van Essen, 1991; Denève et al., 2017). These processes proceed in a *lifelong manner* because knowledge construction is affected by previous experiences. In addition, humans can refine or transfer knowledge acquired from different types of previous tasks built in an incremental manner (Preston and Eichenbaum, 2013; Davachi and DuBrow, 2015). In contrast to human intelligence, ANNs have *catastrophic forgetting* in which the collected information is lost after training of subsequent tasks (Goodfellow et al., 2013). Moreover, the human visual system shows robust performances even in limited data recognition, such as long-tailed and few-shot visual recognition. Real-world data commonly follow long-tailed distribution wherein the majority classes occupy the significant part of the dataset and have an open-ended distribution (Liu et al., 2019b). The primary purpose of long-tailed recognition is to correctly classify the minority class samples to the corresponding targets, reducing the classification bias effect (Cao et al., 2019). Further, the classification of tail class samples can be regarded as a few-shot recognition problem as the degree of imbalance increases (Samuel et al., 2021).

The discrepancy in learning performances between humans and ANNs is closely related to the characteristics of the human brain. First, the human brain operates under two properties: plasticity and stability (Takesian and Hensch, 2013). Plasticity refers to the brain's change in connectivity and circuitry that enables humans to acquire knowledge, keep memories, and adapt to the external environment (Power and Schlaggar, 2017). Meanwhile, stability refers to the ability of long-term memory where stable memory is relevant to stable neuron connectivity (Susman et al., 2019). A balance between plasticity and stability is achieved with excitatory and inhibitory circuit activity in the visual cortex (Takesian and Hensch, 2013). Second, the brain engages the hippocampus and neocortex, as explained by the complementary learning system theory that characterizes learning in the brain (Preston and Eichenbaum, 2013). The hippocampus focuses on acquiring new knowledge, and knowledge is transferred and generalized to the neocortex via the memory consolidation process. Such mechanisms do not exist in backpropagation. However, they can be indirectly performed in learning predictive coding through the free-energy minimization process of predictive coding. As such, we assume that humans can achieve superior performance in MCTs.

## 3. Predictive Coding Networks

Most architectures in ANNs follow an *L*-layer structure wherein each layer consists of a set of neurons (Rumelhart et al., 1986). The training with the backpropagation algorithm can be explained to minimize a global error generated at the last layer of a network. In the backpropagation algorithm, an activation value of each layer is defined as follows:


(1) $$\begin{array}{l} \hat{v_{0}}=x \end{array}$$

(2) $$\begin{array}{l} \hat{v_{i}}=f\left({\hat{v}}_{i-1};\theta_{i}\right) \end{array}$$

where *i* is the indices of layers, and θ_*i*_ is the parameters of *i*-th layer. The goal of backpropagation algorithm is to minimize a loss function L(ŷ,y) between the ground-truth target *y* and the prediction value ŷ. The final layer output is derived from the forward pass as follows:


(3) $$\begin{array}{l} ŷ=f\left(x;\theta \right)={\hat{v}}_{L} \end{array}$$

In the backward pass, the optimization of parameters is performed by the derivative of the loss function. The gradient of each layer is computed in reverse order as follows:


(4) $$\begin{array}{l} \delta_{i}=\delta_{i+1}\frac{\partial f_{i+1}\left({\hat{v}}_{i};\theta_{i+1}\right)}{\partial {\hat{v}}_{l}} \end{array}$$

and


(5) $$\begin{array}{l} d\theta_{i}=-\frac{\partial L\left(ŷ,y\right)}{\partial \theta_{i}} \end{array}$$

where δ_*i*_ and *dθ*_*i*_ are the error signal and the gradient from *i*-th layer, respectively.

**Figure 1:**
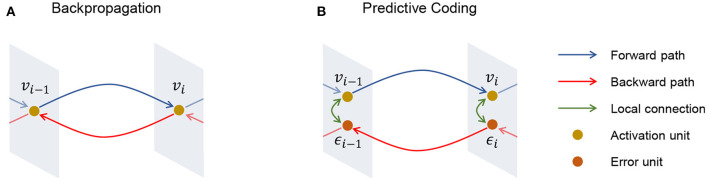
Illustration of **(A)** backpropagation and **(B)** predictive coding. Different from backpropagation, predictive coding has an error unit ϵ_*i*_ for each activation unit *v*_*i*_ and this enables predictive coding to perform local learning.

Meanwhile, in the predictive coding algorithm, an error node is defined in every layer, and the goal of learning is to minimize the collective energy function (Friston, 2003; Bogacz, 2017; Buckley et al., 2017), which is the sum of prediction errors as illustrated in Figure 1. A predictive coding network assumes the network as a directed acyclic computational graph G={E, V} to deliver an error from the last layer to the first layer. E and V are defined as a set of error nodes $e_{i}\in E$ and a set of activation nodes $v_{i}\in V$ at every layer.

By analogy to the cortical hierarchy in the human brain, predictive coding can be formulated as a variational inference algorithm (Friston, 2005; Buckley et al., 2017). Millidge et al. (2020) extended predictive coding to an arbitrary computational graph G considering its hierarchical and generative structure. Given a computational graph G, the feedforward prediction is defined as $p(v_{i})=\Pi_{i}^{N}p(v_{i}|P_{i}))$ and variational posterior is derived as $Q\left(\left\{v_{i}\right\}\right)=\Pi_{i}^{N}Q\left(v_{i}\right)$, where P(x) indicates the set of parent nodes and C(x) denotes the set of child nodes for the given node *x*. Each activation node has the prediction ${\hat{v}}_{i}=f\left(P\left(v_{i}\right);\theta_{i}\right)=f\left({\hat{v}}_{i-1};\theta_{i}\right)$ for *i*-th layer. Based on this, Millidge et al. (2020) defined a objective function of predictive coding as the variational free energy F as follows (Friston, 2005; Buckley et al., 2017):


(6) $$\begin{array}{l} F=KL\left[\left(Q\left(\left\{v_{i}\right\}\right)||p\left(\left\{v_{i}\right\}\right)\right)\right]\geq KL\left[Q\left(\left\{v_{i}\right\}\right)||p\left(\left\{v_{1:N-1}|v_{0},v_{N}\right\}\right)\right]\\ \;\approx \overset{N}{\underset{i=0}{\sum }}e_{i}^{T}e_{i} \end{array}$$

where a prediction error of each layer *e*_*i*_. The *i*-th error node *e*_*i*_ can be calculated as follows:


(7) $$e_{i}={\hat{v}}_{i-1}-v_{i}=f_{i}(v_{i-1};\theta_{i})-v_{i}$$

where *v*_*i*−1_ is the activation node value of the previous layer.

In the backward phase of predictive coding, network parameters θ containing activation nodes {*v*_*i*_} and error nodes {*e*_*i*_} are updated via gradient descent of each layer as follows:


(8) $$\begin{array}{l} dv_{i}=-\frac{\partial F}{\partial v_{i}}=e_{i}-\underset{j\in C\left(v_{i}\right)}{\sum }\frac{\partial \hat{v_{j}}}{\partial v_{i}}. \end{array}$$

The learning is performed by minimizing the variational free energy F until converges as follows:


(9) $$\begin{array}{l} \theta_{i}=\theta_{i}+\eta d\theta_{i} \end{array}$$

where η is the weight learning rate. Parameters are updated as follows:


(10) $$\begin{array}{l} d\theta_{i}=-\frac{\partial F}{\partial \theta_{i}}=-e_{i}\frac{\partial f_{i}\left(v_{i-1};\theta_{i}\right)}{\partial \theta_{i}} \end{array}$$

The equation 10 indicates the local learning rule of the predictive coding where the parameters of *i*-th layer are only updated based on the *e*_*i*_ and *v*_*i*−1_.

## 4. Incremental Learning with Predictive Coding

Based on previous studies (Hassabis et al., 2017; Perez-Nieves et al., 2021), our fundamental assumption is that the more biologically plausible the learning algorithm, closely replicating the learning mechanism of the brain, the more effective it will be for MCTs. Previous studies focused on confirming that the predictive coding network itself inherits the physiological characteristics of the brain. Motivated by the previous study, the current research assumed that predictive coding networks have a latent ability to consolidate the sequentially acquired knowledge in the human memory system. Therefore, we propose a predictive coding framework for incremental learning and verify the efficacy of MCTs. The task of incremental learning can be mainly categorized into two categories (Masana et al., 2020): class-incremental learning and task-incremental learning. The current study focused on the former. In class-incremental learning, the knowledge from previously seen classes is no longer available when a network learns the knowledge of unseen classes, and the learned network aims to achieve favorable classification accuracy for all tasks without forgetting. Multiple tasks were sequentially learned based on the pre-defined order to validate our assumption, and each task with its validation set finishing the training of the given task was evaluated. The algorithms are detailed in Algorithm 1.

**Algorithm 1:** Predictive Coding for Incremental Learning.

Input: Dataset $D_{t=1}^{T}$, Computational Graph G={E,V}, inference learning rate η_*v*_, weight learning rate η_θ_

for all dataset for each task $D_{t}\in D$ **do** ⊳ For each minibatch in the sequential tasks
${\hat{v}}_{0}\leftarrow x_{t}$ ⊳ Initialize the graph with inputs
for all ${\hat{v}}_{i}\in V$ **do** ⊳ Forward phase: calculate predictions
${\hat{v}}_{i}\leftarrow f\left(P\left({\hat{v}}_{i}\right);\theta \right)$
end **for**
ϵ_*L*_←*f*_*L*_(*v*_*L*−1_; θ_*i*_)−*v*_*L*_ ⊳ Compute output error
while *not converged* **do** ⊳ Backward phase: backward iteration
for all $\left(v_{i},\epsilon_{i}\right)\in G$ **do**
$\epsilon_{i}\leftarrow {\hat{v}}_{i-1}-v_{i}$ ⊳ Compute prediction errors
$v_{i}\leftarrow v_{i}+\eta_{v}\frac{dF}{dv_{i}}$ ⊳ Update the vertex values
end **for**
end **while**
end **for**
for all $\theta_{i}^{t}\in E$ **do** ⊳ Update weights at equilibrium
$\theta_{i}^{t}\leftarrow \theta_{i}^{t+1}+\eta_{\theta }\frac{dF}{d\theta_{i}}$
end for **return** θ^*t*^

### 4.1. Experimental Settings

A 3-layer predictive coding network with ReLU non-linearity, where the number of the hidden nodes was 800 for the simple dataset such as MNIST (LeCun et al., 1998) and FMNIST (Xiao et al., 2017), was employed. Similar to the study by Serra et al. (2018), a simplified Alexnet architecture (Krizhevsky et al., 2012) consisting of three convolutional layers was used for the complex dataset such as CIFAR-10 (Krizhevsky et al., 2009). The three convolutional layers comprised 64, 128, and 256 channels.

We refined the data to formulate sequential incremental tasks. The data were divided into multiple portions following the representative incremental learning approaches (Lee et al., 2017; Sokar et al., 2021), and constructed four datasets: disjoint-MNIST, disjoint-FMNIST, split-MNIST, and split-CIFAR-10. Disjoint-MNIST and disjoint-FMNIST were organized by separating MNIST and FMNIST into two tasks. In addition, a more complex dataset, called split-MNIST and split-CIFAR-10, was also established, where all classes were separated into five tasks, and each task contained two categories. The details of the tasks on the multiple datasets are described in Tables 1 and 2. Finally, we evaluated incremental learning performance. We trained a network with sequential order and measured that the acquired knowledge was maintained after each task's training, same as Serra et al. (2018).

**Table 1:** Details of the tasks in the disjoint-MNIST and disjoint-FMNIST benchmarks.

**Task id**	**MNIST classes**	**FMNIST classes**	**Training**	**Testing**
1	[0, 1, 2, 3, 4]	[T-shirt/top, Trouser, Pullover, Dress, Coat]	25000	5000
2	[5, 6, 7, 8, 9]	[Sandal, Shirt, Sneaker, Bag, Ankle boot]	25000	5000

**Table 2:** Details of the tasks in the split-CIFAR-10 benchmark.

**Task id**	**CIFAR-10 classes**	**Category**	**Training**	**Testing**
1	[airplane, car]	vehicle	10000	2000
2	[bird, cat]	animal	10000	2000
3	[deer, dog]	animal	10000	2000
4	[frog, horse]	animal	10000	2000
5	[ship, truck]	vehicle	10000	2000

A learning rate of 0.05 was used, and the learning rate was divided by 1/3 to perform incremental learning, if there was no advancement in the validation loss for five consecutive epochs. In predictive coding, the weight learning rate was set as 0.1 while keeping the other hyperparameters. The minimum learning rate was set as 1*e*^−4^ and batch size as 64. All experiments were conducted using data split according to five different seeds. We provide the code to reproduce the results in the manuscript at https://github.com/jangho2001us/PredictiveCoding_IncrementalLearning.

### 4.2. Experiments on Incremental Learning

Incremental learning was performed on disjoint-MNIST and disjoint-FMNIST using the predictive coding framework to validate our hypothesis. To implement the incremental learning task in a predictive coding manner, we integrated the code of Serra et al. (2018) and Rosenbaum (2021) by replacing the network learning from the backpropagation with the predictive coding networks. The performance of each task was evaluated after completing the learning of each task in Tables 3 and 4. The performance in all tasks learned was evaluated using the best model of the last task. In this case, the best model refers to the model with the highest performance in the given task. Moreover, the other backpropagation-based incremental approaches containing SGD (Goodfellow et al., 2013), SGD-F (Goodfellow et al., 2013), EWC (Kirkpatrick et al., 2017), IMM (Lee et al., 2017), LFL (Jung et al., 2016), and LWF (Li and Hoiem, 2017) were evaluated to observe whether the predictive coding framework itself is effectual for preventing catastrophic forgetting. For all datasets, the average performance of the network trained with SGD based on the predictive coding manner outperformed the performance of the network trained with SGD based on backpropagation. Furthermore, learning with predictive coding exceeds strong competitor EWC (Kirkpatrick et al., 2017) on disjoint-MNIST and split-MNIST.

**Table 3:** Comparison of incremental learning performance (%) on disjoint-MNIST.

**Algorithm**	**Method**	**Task1**	**Task2**	**Average**
BP	SGD (Goodfellow et al., 2013)	88.19	98.99	93.59
	SGD-F (Goodfellow et al., 2013)	99.61	84.56	92.09
	EWC (Kirkpatrick et al., 2017)	92.29	98.99	95.64
	IMM-MEAN (Lee et al., 2017)	98.22	97.10	97.66
	IMM-MODE (Lee et al., 2017)	85.51	98.47	91.99
	LFL (Jung et al., 2016)	93.20	65.78	79.49
	LWF (Li and Hoiem, 2017)	99.43	98.84	99.13
PC	SGD (Goodfellow et al., 2013)	92.80	98.91	95.85

We denoted the learning with backpropagation as BP and learning with the predictive coding framework as PC. We used the five random seeds in the experiments and reported the average performance between task1 and task2.

**Table 4:** Comparison of incremental learning performance (%) on disjoint-FMNIST.

**Algorithm**	**Method**	**Task1**	**Task2**	**Average**
BP	SGD (Goodfellow et al., 2013)	67.37	97.47	82.42
	SGD-F (Goodfellow et al., 2013)	91.87	82.06	86.96
	EWC (Kirkpatrick et al., 2017)	88.79	96.66	92.72
	IMM-MEAN (Lee et al., 2017)	85.70	95.46	87.78
	IMM-MODE (Lee et al., 2017)	64.15	96.33	80.24
	LFL (Jung et al., 2016)	79.00	83.01	81.00
	LWF (Li and Hoiem, 2017)	91.24	97.35	94.30
PC	SGD (Goodfellow et al., 2013)	75.68	97.11	86.40

We denoted the learning with backpropagation as BP and learning with the predictive coding framework as PC. We used the five random seeds in the experiments and reported the average performance between task1 and task2.

**Figure 2:**
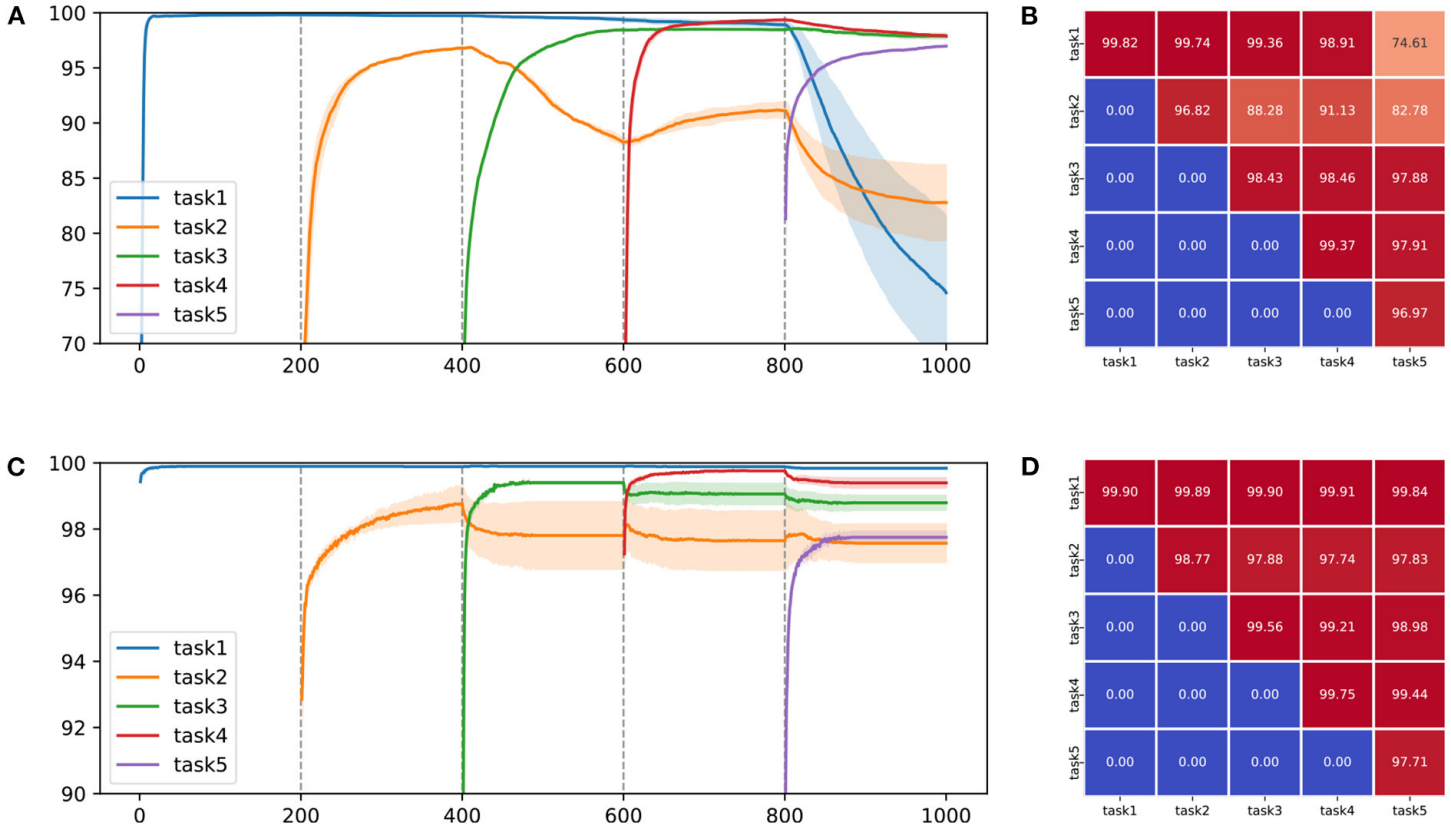
Qualitative and quantitative performance comparison on two learning schemes for **(A,B)** backpropagation and **(C,D)** predictive coding on split-MNIST. In **(A,C)**, the solid line indicates the average accuracy for each task and the transparent region represents the standard deviation on five random seeds. The vertical dashed line refers to the point at which the task to be learned changes. In **(B,D)**, each value indicates the performance each task measured by the final model.

To make the challenging experimental settings, we combined two classes into one task and created five tasks using MNIST and CIFAR-10, similar to the study by Sokar et al. (2021). Incremental learning performance of backpropagation and predictive coding on split-MNIST and split-CIFAR-10 is shown in Tables 5 and 6. The performance of incremental learning based on predictive coding was also compared with that of conventional approaches (Goodfellow et al., 2013; Jung et al., 2016; Kirkpatrick et al., 2017; Lee et al., 2017; Li and Hoiem, 2017). To observe its ability to retain previously obtained knowledge, we visualized the average accuracy of trained tasks in Figure 2. Figure 2 and Table 5 are the experimental results from the same protocol (split-MNIST). After finishing every epoch, we evaluated the performance of all the tasks and drew Figure 2. While Table 5 shows the results of the average evaluation five times using the best model derived from each task. It was confirmed that catastrophic forgetting occurred in both learning algorithms, but the degree of forgetting was certainly more severe in the experimental results of backpropagation. Learning with predictive coding showed stable performance even when the learning task changed, in contrast to the pattern of backpropagation. In the backpropagation experiment, when the network acquired the knowledge of task 3, the knowledge of task 2 was forgotten. Further, when the network learned knowledge of task 5, it was confirmed that the discriminative information of tasks 1 and 2 was removed from the memories. These experimental results confirm that a biologically plausible learning algorithm reduces catastrophic forgetting in incremental learning and enhances the performance of incremental learning as one of MCTs.

**Table 5:** Comparison of incremental learning performance (%) on split-MNIST.

**Algorithm**	**Method**	**Task1**	**Task2**	**Task3**	**Task4**	**Task5**	**Average**
BP	SGD (Goodfellow et al., 2013)	98.52	74.06	93.74	96.43	99.61	92.47
	SGD-F (Goodfellow et al., 2013)	99.95	90.52	95.43	98.06	87.38	94.27
	EWC (Kirkpatrick et al., 2017)	99.41	75.24	94.21	96.34	99.60	92.96
	IMM-MEAN (Lee et al., 2017)	99.94	98.67	94.38	96.55	88.33	95.57
	IMM-MODE (Lee et al., 2017)	99.88	74.20	95.27	97.47	99.42	93.25
	LFL (Jung et al., 2016)	94.34	52.62	54.34	70.63	89.36	72.26
	LWF (Li and Hoiem, 2017)	99.95	99.10	99.77	99.83	99.76	99.68
PC	SGD (Goodfellow et al., 2013)	99.89	97.09	99.28	99.39	98.37	98.80

We denoted the learning with backpropagation as BP and learning with the predictive coding framework as PC. We used the five random seeds in the experiments and reported the average performance from task1 to task5.

**Table 6:** Comparison of incremental learning performance (%) on split-CIFAR-10.

**Algorithm**	**Method**	**Task1**	**Task2**	**Task3**	**Task4**	**Task5**	**Average**
BP	SGD (Goodfellow et al., 2013)	72.17	66.08	71.44	84.17	93.71	77.51
	SGD-F (Goodfellow et al., 2013)	95.72	67.96	60.03	69.97	77.38	74.15
	EWC (Kirkpatrick et al., 2017)	72.76	64.90	67.53	73.99	72.15	70.26
	IMM-MEAN (Lee et al., 2017)	89.71	78.35	78.51	74.73	78.91	80.04
	IMM-MODE (Lee et al., 2017)	76.14	67.07	73.63	84.79	93.87	79.10
	LFL (Jung et al., 2016)	71.50	59.30	71.71	84.47	84.85	74.37
	LWF (Li and Hoiem, 2017)	76.95	70.58	78.46	94.34	93.99	82.86
PC	SGD (Goodfellow et al., 2013)	70.42	74.27	80.70	87.21	90.96	80.71

We denoted the learning with backpropagation as BP and learning with the predictive coding framework as PC. We used the five random seeds in the experiments and reported the average performance from task1 to task5.

We carried out additional experiments to demonstrate the advantages of learning with the brain-inspired algorithm. We implemented the predictive coding version of EWC (Kirkpatrick et al., 2017), IMM-MEAN (Lee et al., 2017), and IMM-MODE (Lee et al., 2017) algorithms and evaluated their performance on disjoint-MNIST. In the EWC algorithm, learning with predictive coding improves the average performance from 95.64% to 97.52%. In addition, learning with predictive coding enhances the average performance 0.21% and 5.42% in IMM-MEAN and IMM-MODE, respectively.

## 5. Limited Data Recognition with Predictive Coding

The potential of predictive coding networks for limited data recognition was then investigated. Specifically, the efficacy of predictive coding networks in long-tailed recognition and few-shot recognition type of MCTs was analyzed. First, real-world datasets are often highly imbalanced following long-tail distribution in which data category accounts for a significant portion of the overall data (Johnson and Khoshgoftaar, 2019; Liu et al., 2019b). Owing to the skewed class distribution of the dataset, the network trained with a class-imbalanced dataset may show a classification bias problem in which the samples of tail classes are predicted as head classes (Cao et al., 2019). In addition, managing few-shot samples in an open-world setting is crucial because it is similar to the situation in which the human recognition system can be encountered. Second, to achieve more human-like recognition performance, effectively managing few-shot examples in an open-world setting is crucial. Two experimental scenarios are significant because it is realistic situations that human recognition can encounter.

The cortical neuron in the human brain can learn with only a few repetitions owing to the local synaptic plasticity (Yger et al., 2015), and it is widely known that such plasticity contributes to the interactions between limited data (Wu et al., 2022). It has been demonstrated that the changes in synaptic connections assist in learning new information and long-term memory formation (Yang et al., 2009). Given the characteristics of synaptic plasticity, experiments with a predictive coding framework were performed on the class-imbalanced data, and the biologically plausible learning algorithm that helped limited data recognition was identified.

### 5.1. Experimental Settings

The same architecture used in the previous section consisting of three-layer MLP was used in long-tailed recognition. The number of hidden neurons was set as 800 with ReLU non-linearity and dropout. We used MNIST (LeCun et al., 1998) for our experiment and synthesized the long-tailed data with an imbalance ratio γ. The imbalance ratio was defined as the proportion of the samples of the highest number of classes to the lowest number of classes as $\frac{N_{max}}{N_{min}}$. Although it differed depending on the imbalance ratio, in general, *N*_*max*_ and *N*_*min*_ usually followed the relationship, *N*_*max*_≫*N*_*min*_. Exponential distribution and the number of samples *N*_*l*_ in *l*-th class was defined as $N_{l}=N_{max}\cdot \gamma^{-\frac{l-1}{L-1}}$. The four types of imbalanced data distribution were then synthesized as previously described (Kim et al., 2020). To train a network, we set a batch size of 128 and optimized a model until 100 epochs. When backpropagation was used for learning, the learning rate was increased from 0.0001 to 0.5 by growing five times, and the best performance results among them were determined. When predictive coding was used for the optimization, a learning rate of 0.002 with a weight decay of 2*e*^−4^ was used. Additionally, the weight learning rate η was set as 0.1 and the number of iterations as 20 as hyperparameters for predictive coding networks. All the experiments with predictive coding were performed under the fixed prediction assumption. We provide the code to reproduce the results in the manuscript at https://github.com/jangho2001us/PredictiveCoding_LongTailedRecognition.

In few-shot recognition, the same experimental settings with those of Snell et al. (2017), which comprised four convolutional blocks with Batch normalization, ReLU, and MaxPool were used. Experiments on few-shot recognition were conducted with Omniglot (Lake et al., 2011) dataset containing 1623 categories of handwritten characters. The performance of few-shot recognition is commonly measured by *N*-way *k*-shot classification, where *N* implies the number of given classes and *k* indicates the number of samples in each category. The current study extended the experimental protocol of the original paper to 30-way *k*-shot experiment settings because those evaluation protocols are more difficult because the number of classes for the candidate group increases. The learning rate was set to 1*e*^−3^ and then reduced by 1/10 every 20 epoch to train a network. For learning networks with a predictive coding framework, the same learning rate, weight decay, weight learning rate, and iterations were used. For more information, please refer to the original paper (Snell et al., 2017). We provide the code to reproduce the results in the manuscript at https://github.com/jangho2001us/PredictiveCoding_FewShotRecognition.

### 5.2. Experiments on Long-tailed Recognition

In Table 7, we compared the long-tailed recognition performance with Cross-Entropy (CE) loss, Mixup approach (Zhang et al., 2017), Focal loss (Lin et al., 2017), Class-Balanced Focal (CB Focal) loss (Cui et al., 2019), Label-Distribution-Aware-Margin (LDAM) loss (Cao et al., 2019), and Balanced Meta-Softmax (BALMS) loss (Ren et al., 2020). Further details on multiple learning objectives are provided in the Supplementary material. The experimental results showed the benefit of learning with predictive coding networks. First, the long-tailed recognition performance was higher by 4.45% in learning the network with a predictive coding framework than that in learning with CE loss under severe class imbalance of data distribution. Similar results in the following experiments were observed when the network was trained with other learning objectives such as Focal (Lin et al., 2017) and BALMS (Ren et al., 2020). In this experiment, the performance improvement is evaluated using the predictive coding framework rather than comparing performance between different learning objectives. The results shown in Table 7 indicate that the learning algorithm close to the human brain brings a positive effect on MCTs, confirming our assumption.

**Table 7:** Comparison of classification performance (%) on MNIST under four different imbalance distributions.

		**Imbalance Ratio (**γ**)**			
**Algorithm**	**Objective Function**	**200**	**100**	**50**	**10**
BP	CE	68.78	78.06	89.63	97.17
	Mixup (Zhang et al., 2017)	67.60	76.69	86.97	96.15
	Focal (Lin et al., 2017)	70.92	79.42	90.89	97.31
	CB Focal (Cui et al., 2019)	69.93	79.72	91.26	97.09
	LDAM (Cao et al., 2019)	65.17	75.58	84.91	97.14
	BALMS (Ren et al., 2020)	72.25	81.34	92.50	97.23
PC	CE	73.23 (+4.45)	79.26 (+1.20)	90.10 (+0.47)	97.37 (+0.20)
	Mixup (Zhang et al., 2017)	67.77 (+0.17)	77.60 (+0.91)	88.26 (+1.29)	96.27 (+0.12)
	Focal (Lin et al., 2017)	71.99 (+1.07)	79.57 (+0.15)	91.18 (+0.29)	97.03 (-0.28)
	CB Focal (Cui et al., 2019)	70.19 (+0.26)	80.28 (+0.56)	91.40 (+0.14)	97.24 (+0.14)
	LDAM (Cao et al., 2019)	65.54 (+0.37)	76.05 (+0.47)	85.08 (+0.17)	97.20 (+0.06)
	BALMS (Ren et al., 2020)	74.22 (+1.97)	82.28 (+0.94)	93.50 (+1.00)	97.45 (+0.22)

Experiments are performed with five random seeds, and the average performance is reported. Relative variance is provided in the bracket. Increments are presented as red and decrements as blue.

### 5.3. Experiments on Few-shot Recognition

The few-shot recognition performance trained with backpropagation and predictive coding framework is shown in Table 8, Learning with predictive coding enabled robust recognition under the various few-shot experimental protocols. Additionally, predictive coding networks showed their potential ability under challenging inference settings such as 20-way 1-shot and 30-way 1-shot rather than 20-way 5-shots and 30-way 5-shots. The experimental results confirmed our assumptions and supported that the brain-like learning algorithm was effective for MCTs.

**Table 8:** Experimental results on the low-shot recognition on the Omniglot dataset.

**Algorithm**	**Method**	**5-way Acc**.		**10-way Acc**.		**20-way Acc**.		**30-way Acc**.	
		**1-shot**	**5-shot**	**1-shot**	**5-shot**	**1-shot**	**5-shot**	**1-shot**	**5-shot**
BP	ProtoNet	98.41	99.56	96.87	99.18	94.64	98.54	92.97	97.98
	(Snell et al., 2017)
PC	ProtoNet	98.46	99.59	96.98	99.19	94.88	98.59	93.14	98.05
	(Snell et al., 2017)	(+0.05)	(+0.03)	(+0.11)	(+0.01)	(+0.24)	(+0.05)	(+0.17)	(+0.07)

Five random seeds are used in the experiment, and the average performance is reported. Relative variance is shown in the bracket. Increments are presented as red.

## 6. Discussion

### 6.1. Analysis of Plasticity-stability Aspects

The plasticity-stability dilemma is a well-known problem widely studied in biology (Mateos-Aparicio and Rodŕıguez-Moreno, 2019). This phenomenon is related to the power of consolidation of new information without forgetting previously acquired information (Mermillod et al., 2013). Further, it is an essential issue in incremental learning with ANNs (Lin et al., 2022). The human brain is well-controlled to learn new information and to prevent the learned information from being overridden by the new information (Takesian and Hensch, 2013). However, ANNs naturally induce catastrophic forgetting and expose the trade-off between plasticity and stability (Kirkpatrick et al., 2017).

**Figure 3:**
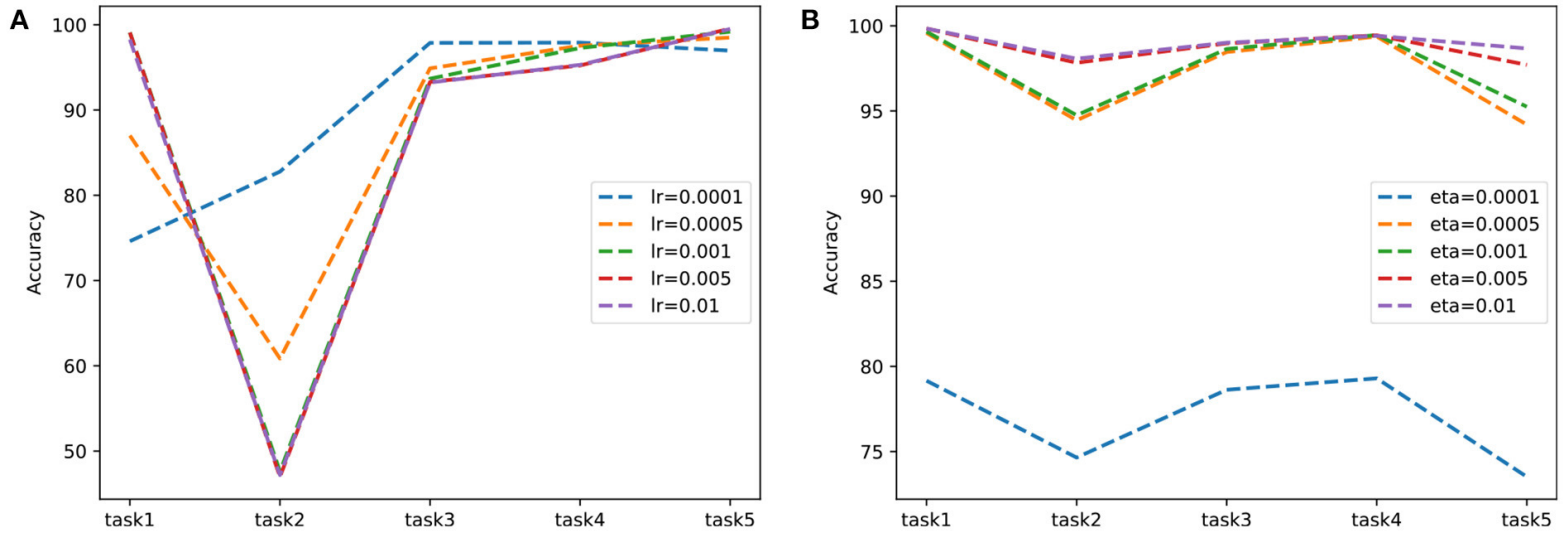
Comparison of learning with **(A)** backpropagation and **(B)** predictive coding on split-MNIST in two learning schemes. To adjust network stability, the learning rate of backpropagation and the weight learning rate of predictive coding are varied.

To confirm that predictive coding achieves a better plasticity-stability trade-off than backpropagation, we experimented with split-MNIST by controlling the stability of two learning mechanisms. Adjusting the learning rate is not directly related to stability, but it was used because it was considered as a factor that could adjust stability in our experiments. In Figure 3, we report the experimental results and compare the learning schemes by adjusting the learning rate of backpropagation and the weight learning rate of predictive coding. In backpropagation experiments, the learning is reduced from 0.01 to 0.0001 to decrease forgetting of acquired knowledge. When the learning rate was 0.0001, the network forgot less information to perform task 2. However, it still showed limited performance in tasks 1 and 2. Thus, maintaining stability by reducing the learning rate may not be acceptable because it deteriorates the overall performance. Meanwhile, performance was consistently high for each task in predictive coding experiments. These results implied predictive coding had better plasticity properties than backpropagation while maintaining stability.

### 6.2. Interplay of Hippocampus and Prefrontal Cortex

The hippocampus plays an essential role in episodic memory at the top of the cortical processing hierarchy (Felleman and Van Essen, 1991). In incremental learning, the ability to regulate learned information and retrieve context-appropriate memories is essential. We can understand the effectiveness of predictive coding in incremental learning as the interaction between the hippocampus and the prefrontal cortex in the human brain (Eichenbaum, 2017; Barron et al., 2020). It is well known that the hippocampus can quickly encode new information, stabilize memory traces, and organize memory networks (Preston and Eichenbaum, 2013). In addition, this mechanism has been physiologically proven through functional magnetic resonance imaging studies (Hindy et al., 2019).

We have shown that the learning process of predictive coding networks is analogous to the interaction between the hippocampus and the prefrontal cortex in the human brain (Eichenbaum, 2017). As described in Algorithm 1, the learning process based on predictive coding networks can be divided into two phases: forward and backward pass. In the forward phase, the predictive coding network computes its predictions for every layer. In the backward phase, the predictive coding network minimizes the free-energy summation as a learning objective. The two-phase learning of predictive coding networks corresponds to acquiring and consolidating information in the hippocampus and prefrontal cortex. The predictive coding framework promotes the two processes and enables accurate inference when data containing information corresponding to the previously learned task are received.

### 6.3. Rationale for Selecting Predictive Coding

The reason why we selected predictive coding as a brain-inspired algorithm is as follows. As described in Section 2, predictive coding is potentially more biologically plausible because local learning rules perform parameter updates. This property is distinct from the update of backpropagation executed from the global error signal. It will be ideal if the parameter update is performed asynchronously in a different layer, such as the neural plasticity of the human brain. However, the parameter update of predictive coding occurs under the *fixed prediction assumption* (Millidge et al., 2020). The fixed prediction assumption implies that the parameters are updated based on the *fixed* predictions of the forward phase. Whittington and Bogacz (2017) demonstrated that a predictive coding network with a fixed prediction assumption performs the same parameter updates as backpropagation. Another limitation of predictive coding is the degree of convergence of variational free energy used as a learning objective. The convergence of the backward phase is achieved by setting a specific number of iterations (Rosenbaum, 2021). Depending on the number of backward iterations, learning with predictive coding may converge or diverge. Although these two issues introduced earlier remain open questions, we conducted our experiments using predictive coding because we thought its advantages outweighed its disadvantages.

## 7. Conclusion

This study empirically demonstrated the potential effectiveness of predictive coding in MCTs. However, despite this, the predictive coding algorithm still has some limitations. First, predictive coding requires a longer training time than backpropagation because it executes backward iteration until the error nodes and activation nodes converge. Although we expanded our experiments for large networks such as VGGNet and ResNet (He et al., 2016; Krizhevsky et al., 2017), we could not perform the experiments on MCTs because of the excessive training time. Second, to conduct learning with predictive coding, the network should be an architecture composed of sequential layers. For example, if shortcut connections exist, it is challenging to implement them into a predictive coding layer. In this case, we set the block unit, which is the boundary of the shortcut, as the predictive coding layer. If predictive coding combines learning speed and scalability, there will be infinite opportunities for development as a learning algorithm that can replace backpropagation.

In summary, we extensively analyze the benefits of learning ANNs with predictive coding frameworks for MCTs. MCTs can be described as tasks that are easy for human intelligence while difficult for machine intelligence. Based on our hypothesis, we empirically demonstrate that brain-inspired predictive coding has advantages in incremental learning on MNIST and CIFAR, long-tailed recognition on MNIST, and few-shot recognition on Omniglot. In neuroscience, especially the intrinsic properties of the human brain, we discuss why training ANNs with a predictive coding framework improves the performance of MCTs. The study concludes that predictive coding learning is similar to the plasticity-stability property of the human brain and mainly mimics the interaction between the hippocampus and prefrontal cortex. Finally, it is an interesting avenue for future work to reduce the training time under the fixed prediction assumption and relax the constraint of predictive coding while maintaining the performance.

## Data availability statement

The original contributions presented in the study are publicly available. The data can be found here: http://yann.lecun.com/exdb/mnist and https://www.cs.toronto.edu/~kriz/cifar.html.

## Author contributions

JL contributed to the design of the study and performed the experiments. JL, JJ, BL, and J-HL developed the algorithm and performed the result analysis, and wrote the revised manuscript. SY conceived and supervised the project, checked ideas and terminology, performed result analysis, editing, and revision of the manuscript.

## Funding

This work was supported by Electronics and Telecommunications Research Institute (ETRI) grant funded by the Korean government [22ZS1100, Core Technology Research for Self-Improving Integrated Artificial Intelligence System], and the BK21 FOUR program of the Education and Research Program for Future ICT Pioneers, Seoul National University in 2022.

## Conflict of interest

The authors declare that the research was conducted in the absence of any commercial or financial relationships that could be construed as a potential conflict of interest.

## Publisher's note

All claims expressed in this article are solely those of the authors and do not necessarily represent those of their affiliated organizations, or those of the publisher, the editors and the reviewers. Any product that may be evaluated in this article, or claim that may be made by its manufacturer, is not guaranteed or endorsed by the publisher.
