# Supplementary material for: Brain-inspired Predictive Coding Improves the Performance of Machine Challenging Tasks
Source: Front Comput Neurosci. 2022 Nov 16;16:1062678. doi: 10.3389/fncom.2022.1062678 (PMC9709416; doi:10.3389/fncom.2022.1062678)
Supplement: Supplementary file 1 [file Data_Sheet_1.PDF]

Brain-inspired Predictive Coding Improves the Performance of Machine Challenging Tasks

1 COMPARISON METHODS FOR INCREMENTAL LEARNING

We provide a brief introduction to the learning algorithms we utilized.

1.1 SGD

We trained each task with pre-defined order using stochastic gradient descent (SGD) (Ruder, 2016). We denoted an approach that froze the parameters except for that of the last layer as SGD-F.

1.2 EWC

To combat the catastrophic forgetting, Kirkpatrick et al. (2017) introduced Elastic Weight Consolidation (EWC) algorithm as a regularization approach. It helps for the posterior probability to maintain the important parameters for the previously learned tasks and jointly preserve the ability to learn new tasks.

1.3 IMM

Lee et al. (2017) introduced a method, called incremental moment matching (IMM) to resolve catastrophic forgetting. To approximate the posterior distribution of parameters, IMM utilizes a Gaussian distribution for each task. The goal of IMM is to investigate for the optimal parameters of the Gaussian approximation and it is categorized into two algorithms. First, IMM-MEAN calculates the weighted average of two networks by minimizing the local KL-divergence. While IMM-MODE focuses on the searching the mode that maximizes the posterior.

1.4 LFL

Jung et al. (2016) proposed a less-forgetting method to alleviate catastrophic forgetting. By constructing source and target networks, the goal of LFL minimize the discrepancy between the feature vectors of from two networks.

1.5 LWF

Li and Hoiem (2017) designed a less forgettable architecture in incremental learning. The architecture is constructed with multiple output layers where each layer is appended when the network learns a new task. To effectively maintain information from previously learned, LWF utilizes knowledge distillation loss.

2 COMPARISON METHODS FOR CLASS-IMBALANCED LEARNING

We provide a brief introduction to the learning objectives we utilized.

2.1 Cross-Entropy Loss

The most common criterion used for classification tasks. Inspired from the information theory, the goal of cross-entropy loss is to make the probability distribution be as close as possible to the target distribution.

2.2 Mixup

Mixup (Zhang et al., 2017) generates synthetic training examples via linear interpolation as follows: $\tilde{x} = \lambda x_i + (1-\lambda)x_j$, and $\tilde{y} = \lambda y_i + (1-\lambda)y_j$, where (x_i, y_i) and (x_j, y_j) are two training examples, and $\lambda \in [0, 1]$ is the mixing coefficient. It is demonstrated that the training with virtual examples enhances the performance of classification and increases the robustness of networks.

2.3 Focal Loss

As a representative technique for skewed data distribution, focal loss (Lin et al., 2017) is designed when the object detector encountered class imbalance. It is implemented by multiplying a re-weighting factor $\alpha(1-p)^\gamma$ on the cross-entropy loss where α indicates the class-wise weight.

2.4 Class-Balanced Focal Loss

Class-balanced loss (Cui et al., 2019) considers the effective number of samples to adjust the decision boundary by inverse class frequency. The effective number for each class is defined as $E_n = \frac{1-\beta^n}{1-\beta}$ and it is multiplied by the cross-entropy loss.

2.5 Label Distribution Aware Margin Loss

Cao et al. (2019) proposed a loss function that adjusts the decision boundary of which a class having a small number of samples has a wider margin to effectively handle the imbalanced data and a re-balancing scheduler for the efficient optimization of the re-weighting strategy.

2.6 Balanced Meta-SoftMax Loss

Ren et al. (2020) focused on the distribution mismatch between training and testing dataset, and propose a balanced softmax. However, due to the property of mini-batch training, the balanced sofmax does not showed successful performance due to the over-balance problem. To resolve this problem, Ren et al. (2020) introduced a meta sampler which is a trainable version of a class-balanced sampler.

REFERENCES

Cao, K., Wei, C., Gaidon, A., Arechiga, N., and Ma, T. (2019). Learning imbalanced datasets with label-distribution-aware margin loss. *Advances in neural information processing systems* 32

Cui, Y., Jia, M., Lin, T.-Y., Song, Y., and Belongie, S. (2019). Class-balanced loss based on effective number of samples. In *Proceedings of the IEEE/CVF conference on computer vision and pattern recognition*. 9268–9277

Jung, H., Ju, J., Jung, M., and Kim, J. (2016). Less-forgetting learning in deep neural networks. *arXiv preprint arXiv:1607.00122*

Kirkpatrick, J., Pascanu, R., Rabinowitz, N., Veness, J., Desjardins, G., Rusu, A. A., et al. (2017). Overcoming catastrophic forgetting in neural networks. *Proceedings of the national academy of sciences* 114, 3521–3526

Lee, S.-W., Kim, J.-H., Jun, J., Ha, J.-W., and Zhang, B.-T. (2017). Overcoming catastrophic forgetting by incremental moment matching. *Advances in neural information processing systems* 30

Li, Z. and Hoiem, D. (2017). Learning without forgetting. *IEEE transactions on pattern analysis and machine intelligence* 40, 2935–2947

Lin, T.-Y., Goyal, P., Girshick, R., He, K., and Dollár, P. (2017). Focal loss for dense object detection. In *Proceedings of the IEEE international conference on computer vision*. 2980–2988

Ren, J., Yu, C., Ma, X., Zhao, H., Yi, S., et al. (2020). Balanced meta-softmax for long-tailed visual recognition. *Advances in neural information processing systems* 33, 4175–4186

Ruder, S. (2016). An overview of gradient descent optimization algorithms. *arXiv preprint arXiv:1609.04747*

Zhang, H., Cisse, M., Dauphin, Y. N., and Lopez-Paz, D. (2017). mixup: Beyond empirical risk minimization. *arXiv preprint arXiv:1710.09412*
